# Annotations

**Published:** 1899-07-01

**Authors:** 


### Annotations.

### The Resistance of Bacteria to Cold.

In a paper read recently before the Philadelphia Pathological Society Dr. Mazyck P. Ravenel[1] describes a series of experiments which he had made with liquid air with the object of ascertaining the behaviour of bacteria in the presence of intense cold. He says that the temperature of liquid air is 312 deg. F. below zero, and that when one sees the effect it has upon inanimate objects, and the rapidity with which it destroys tissues, one finds it hard to believe that vegetable life can withstand it even for a few moments. Yet, he adds, such is the case. His experiments were made with anthrax spores dried on silk threads, with recently-grown cultures of diphtheria on blood serum, and with agar cultures of the typhoid fever bacillus and the bacillus prodigiosus. In the latter three cases a suspension of the growth was made in sterile water with which the threads were impregnated, and these were then immersed in the liquid air without drying. Anthrax spores were exposed to the action of liquid air for from one minute to three hours; the diphtheria bacillus up to 30 minutes; and the typhoid bacillus and the prodigiosus up to 60 minutes; after which they were transferred to bouillon and placed in the incubator. In no case were the bacteria killed, nor could any inhibition even be detected, all cultures growing as rapidly and as vigorously as the controls. One need hardly say that the matter is one of very considerable interest as bearing upon the question how far the maintenance of vitality—as, for example, in seeds and resting spores—is necessarily attended with a continuance of metabolism and tissue waste. Much laughter has been from time to time indulged in at the expense of those who have asserted that they have raised wheat from seeds found in the tombs of the Pharaohs after having been bottled up for several thousand years. But after all it is as difficult to conceive of vital processes going on at a temperature of 312 deg. F. below zero as it is to imagine the same vital process of merely "keeping alive," persisting for some thousands of years on the very small amount of pabulum accessible to the ovum. It is an old question enough. Does the maintenance of life demand the unbroken continuance of life's processes, with all their wear and tear, in however infinitesimal a degree? or does life depend on mere structure, and may it, under certain conditions and at certain points in its history, be held in abeyance so long as structure is not interfered with, and be started afresh in full activity when the proper surroundings are once again restored?

[1] Med. News, June 10.

### Holidays in the Alps.

When people are looking forward to their holidays and are working out that most interesting problem "where to go," it is not unnatural that they should look back upon past seasons and consider what places have, as the saying is, "suited them" best on previous occasions. Unfortunately, experience in such matters is far from being a trustworthy guide. Each man thinks that he knows himself, and that his personal experience of his own powers will best tell him what he can do, but he too often forgets that his own experience is necessarily that of a man younger than he now is, and that when he arrives at the age when arteries are getting stiff and unable to accommodate themselves to varying conditions, the experience of more youthful days is absolutely misleading. This is of peculiar importance in the case of those who have got into the habit of going up year after year into the mountains. That condition of arterio-sclerosis which makes the mountains dangerous is apt to creep on very insidiously, and to produce the very sensations of lack of tone which a man's own experience has taught him is best relieved by a run among the glaciers. Hence come accidents which ought to be avoided. Where there is arterial sclerosis, although the heart may have become fairly able to meet the increased strain upon it under accustomed conditions, neither heart nor arteries may be able to accommodate themselves quickly to new surroundings, and the dangers of high altitudes in such cases are greatly increased by the modern facilities for reaching them with extreme rapidity. If in old days an invalid loitering on the lower levels in Switzerland had felt any hankering after the hills he would have been restrained by the labour of getting there, and at the worst he would have crept up slowly on some patient animal, and thus would have become gradually accustomed to the change. Not so nowadays. The ever-present "funicular" whisks one up to unknown altitudes in no time. It costs but a few pence. One sits quietly in a railway carriage instead of being shaken to pieces on an uneasy steed. Nothing can seem simpler or more free from fatigue. But the heart and circulation find it out. The lesson we wish then to insist upon is that where the vessels are rigid and the heart is weak, and especially where there are suspicions of imperfect kidneys, sudden changes of altitude are often badly borne, notwithstanding that the patient's own experience, gathered in earlier years, may lead him to assert and to believe that all he wants is "a whiff of mountain air."

### Milk from the Aldershot Sewage Farm.

Complaints having been made that the milk supplied to the troops at Aldershot was of an unhealthy or dangerous character, it being the product of cows fed on the produce of the Aldershot Sewage Farm, the War Office authorities, while maintaining that there were no proper grounds for such complaints, allowed the matter to be submitted to an independent sanitary authority. Dr. Andrewes, of St. Bartholomew's Hospital, was, therefore, requested to examine the conditions of the sewage farm in question, and to advise whether these are such as to throw any doubts upon the safety of making use of the milk produced upon the farm for human consumption. After a complete investigation Dr. Andrewes supports the military authority in considering the use of the milk free from risk. We think that it is pretty well understood now that the dairy products obtained from a well-managed sewage farm are not injurious to health. But it must be insisted on that all depends upon the meaning of the phrase "well managed," so that while we quite accept what Dr. Andrewes has said, as deciding the point for the present, we cannot take it as having much force in regard to the future. The Aldershot Sewage Farm has had a bad sanitary reputation for a long time, and the repentance of a scapegrace, even though testified to by good works, cannot be taken as proof of permanent reformation.

